# Antimicrobials administration time in patients with suspected sepsis: is faster better? An analysis by propensity score

**DOI:** 10.1186/s40560-020-00448-1

**Published:** 2020-04-22

**Authors:** Johana Ascuntar, Deibie Mendoza, Fabián Jaimes

**Affiliations:** 1 GRAEPIC—Clinical Epidemiology Academic Group (Grupo Académico de Epidemiología Clínica), the University of Antioquia, Medellín, Colombia; 2 University of Antioquia, Medellín, Colombia; 3 Department of Internal Medicine, University of Antioquia, Medellín, Colombia; 4 Hospital San Vicente Fundación, Medellín, Colombia

**Keywords:** Sepsis, antimicrobials, antibiotics, propensity score, in-hospital mortality

## Abstract

**Background:**

Early use of antimicrobials is a critical intervention in the treatment of patients with sepsis. The exact time of initiation is controversial and its early administration may be a difficult task in crowded emergency departments (ED). The aim of this study was to estimate, using a matched propensity score, the effect on hospital mortality of administration of antimicrobials within 1 or 3 hours, in patients admitted to the ED with sepsis.

**Methods:**

This was a secondary analysis of a multicenter prospective cohort. Patients included in the study were older than 18 years, hospitalized between 2014 and 2016 with suspected sepsis, and admitted to ED of three tertiary care university hospitals in Medellín, Colombia. A propensity score analysis for administration of antimicrobials, both within 1 and 3 h of admission by the ED, was fitted with 28 variables related with clinical attention and physiological changes. As a sensitivity analysis, a logistic regression model was fitted for antimicrobial use adjusted both by propensity score and confounding variables.

**Results:**

The study cohort was composed of 2454 patients with a median age of 62 years (IQR = 46–74). Among them, 32% (*n* = 781) received antibiotics within 3 h and 14% (*n* = 340) within the first hour. The main diagnoses were urinary tract infection (28%, *n* = 682) and pneumonia (27%, *n* = 671). Blood cultures were obtained in 87% (*n* = 2140) and yielded positive in 29% (*n* = 629), mainly with *Escherichia coli* (37%, *n* = 230), *Staphylococcus aureus* (21%, *n* = 132), and *Klebsiella pneumoniae* (10.2%, *n* = 64). The hospital mortality rate was 11.5% (*n* = 283). There were no significant differences in mortality, after adjustment, using antimicrobials either in the first hour (OR 1.03; 95% CI = 0.63; 1.70) or 3 h (OR 0.85; 95% CI = 0.61; 1.20). There were no changes with different models for sensitivity analysis.

**Conclusions:**

Despite the obvious constraints given for sample size and residual confounding, our results suggest that we need a more comprehensive approach to sepsis and its treatment, considering early detection, multiple interventions, and goals beyond the simple time-to-antimicrobials.

## Background

In recent years, sepsis has been established as a public health problem with up to 30 million cases per year in the world and approximately five million deaths estimated, although with predominant information from high-income countries [1]. The Surviving Sepsis Campaign has established several pillars in the management of patients with sepsis [2]. Among the guidelines included are the initial resuscitation with intravenous fluids, the obtaining of blood cultures, the measurement of lactate levels, the control of the source of infection, antimicrobial therapy, and the use of vasopressors.

Although compliance with these elements has been associated with a reduction in mortality in patients with sepsis [3], their general implementation has not been without controversy [4, 5]. Particularly, the early administration of antimicrobials in a real-life setting ED presents, among others, logistical difficulties. Controversies persist regarding the optimal time to start antimicrobials (within 1 h, 3 h, or some other time point) and the correct definition of time zero (either after admission to the ED or after recognition of sepsis) [6]; this is because its recognition can be difficult and its specific onset is rarely known. With this in mind, it is important to consider that the indiscriminate use of antimicrobials can favor resistance and the occurrence of adverse effects, this being a potential disadvantage of the early administration of antimicrobials, together with higher inappropriate prescription rates [7].

A study in this field is necessary because the evidence of time-to-antimicrobials that supports the most recent guidelines comes mainly from retrospective studies, due to the ethical and logistical considerations of a clinical trial in the area. Having in mind that some other prospective studies controvert these 1-h guidelines, this investigation seeks to estimate, through the matching of the propensity score [8], a statistical technique that favors the reduction of bias due to prognostic factors [9], the effect of administering antimicrobials within 1 or 3 h on in-hospital mortality of patients admitted to ED with sepsis.

## Materials and methods

### Study and design

Secondary analysis obtained from a prospective cohort study [10]. The purpose of our primary study was to estimate the effect of each component of early goal-directed therapy (EGDT) protocol, as well as the effect of antibiotics use, on in-hospital mortality of patients with septic shock (according to the Sepsis-2 definition). The study was conducted in emergency departments and critical care units of three tertiary care university hospitals in the city of Medellín, Colombia: the Hospital Universitario San Vicente Fundación (HUSVF, 560 adult beds and 45 ICU beds in 4 units), the Hospital Pablo Tobón Uribe (HPTU, 360 adult beds and 40 ICU beds in 3 units), and the IPS Universitaria León XIII (IPSU, 450 adult beds and 24 ICU beds in 2 units). The period of patient recruitment and data collection was between June 2014 and February 2016 [10], and for the current study, we analyzed the total eligible population with any organ dysfunction, in addition to those with septic shock.

### Study population

Inclusion criteria are as follows: patients aged greater than or equal to 18 years old; admitted to the ED with suspected sepsis, septic shock, or a record in the clinical history of infection under the criteria of the Center for Disease Control and Prevention (CDC) [11]; and at least one of the following criteria for organ dysfunction: Glasgow scale < 15; PaO_2_/FiO_2_ index < 300 or the need for mechanical ventilation; urinary output < 0.5 ml/kg/h for 2 h reported in clinical record; creatinine (CR) > 2 mg/dL with no history of previous kidney disease or an 0.5 mg/dl increase over previous values; international normalized ratio (INR) > 1.5 s; partial thromboplastin time (PTT) > 60 s; platelet count (PLT) < 150,000 cells/mm^3^; total bilirubin (T Bil) > 2 mg/dl; lactic acid (LA) > 2 mmol/l; capillary refill time more than 2 s; systolic blood pressure (SBP) < 90 mmHg or mean arterial pressure (MAP) < 70 mmHg during the first 6 h after admission. The current Sepsis-3 criteria were not part of the study population collection process because these were published in late February 2016. However, we analyzed the total eligible population with any organ dysfunction, which is overly similar to the current one based on the SOFA score.

Exclusion criteria are as follows: refusal by the patient, his family, or the attending physician to participate in the study; concurrent diagnoses of pregnancy, myocardial infarction, stroke, asthmatic crisis, arrhythmia, trauma, gastrointestinal bleeding, seizure not due to meningitis, psychoactive substance overdose, surgery < 24 h, burns, CD4 count < 50 cells/mm^3^, hyperosmolar status, diabetic ketoacidosis or cirrhosis; discharge or remission in the first 24 h of hospitalization; prior participation in the study; referral from another institution where they had been hospitalized for more than 24 h or a no-resuscitation order.

### Measurements

#### Exposure variables

The procedures and treatments performed during the first 24 h of hospital stay were recorded, according to the original protocol of Rivers et al. [12]. The administration of antimicrobials was recorded during the first 24 h of hospital stay (antimicrobial administration within 1 h and within 3 h; time zero was defined as admission to the ED, which we consider more relevant from the clinical point of view). The type and dosage of the treatments were taken into consideration: intravenous fluids, antimicrobials, vasopressors, and transfusions. In addition, the researchers evaluated the antimicrobial scheme administered by classifying it as adequate or inadequate, according to the final microbiological isolation or, if this does not exist, according to the clinical criteria in each case.

#### Potential confounding variables

As potential confounding variables were taken into account: age, quantity of intravenous fluids (IVF), blood cultures in the first 3 h, and Charlson Index [13]; the severity of sepsis, assessed using Sepsis-related Organ Failure Assessment (SOFA) score [14] and Acute Physiology and Chronic Health Evaluation (APACHE II) scoring system [15]; and the confirmed diagnosis of infection, the inadequate use of antimicrobials, and the lactate values at admission. The definition of sources of infection was standardized according to the CDC criteria [11]. In the included hospitals, the treatment strategy was mainly determined by the treating physician. Nevertheless, all the institutions had availability of the resources needed to apply the complete treatment and also had a basic suggested protocol that included continuous monitoring, IVF bolus with crystalloid according to the blood pressure and central venous pressure, early administration of adequate antimicrobials in the first 3 h depending on the suspected focus of infection, taking of appropriate cultures and initiation of a vasopressor when blood pressure did not improve with IVF.

#### Primary outcome

In-hospital mortality

### Data source

In the primary study, all patients who presented to the ED with a diagnosis of infection, sepsis, severe sepsis, or septic shock were screened. The source of the infection and the presence of organ dysfunction or shock were verified with the data extracted from the clinical records in the first 6 h. All data on the diagnosis and treatment process, including time, were also taken from the clinical records. Trained research nurses in each institution carried out the entire process of screening, selection, and collection of information by means of a standardized form. The clinical researchers performed a permanent review and verification of the patients included and the data collected. Patients were followed until hospital discharge.

### Statistical analysis

#### Propensity matching

To generate the propensity score (PS) [8], two logistic regression models were made for the use of antimicrobials in the first hour and 3 h after admission to the ED, respectively. To test the fit of the models, we used the adjusted pseudo-*R*^2^ (1 = perfect fit of the data to the model), the Hosmer-Lemeshow goodness-of-fit statistic (*p* > 0.05) and the area under the ROC curve (AUC - ROC Curve) (0.5 = non-discrimination, 1 = perfect discrimination). Under these criteria, the model, which included 28 variables related to the clinical care of the patients as well as direct measurements of physiological derangement, was selected (Additional files 1 and 2), as all of them may prognostic factors associated with both the outcome and the likelihood of being exposed to the intervention [16]. With the PS derived from these variables, the nearest neighbor 1:1 match was used, selecting for each treated individual the control individual with the shortest distance according to local optimal algorithms (greedy algorithms), no caliper and no replacement [17]. A standardized mean difference (SMD) < 10% was considered an adequate balance in the distribution between the groups.

For the evaluation of the pre- and post-matching groups in the continuous variables, median and interquartile ranges were measured, which were compared by means of the Mann-Whitney *U* test; and categorical variables were described with proportions, compared by Pearson's chi-square test.

#### Outcome models

Finally, as a sensitivity analysis, several logistic regression models were performed both in the population matched by propensity score and in the total population. These models were on the general population and in subgroups by (1) confirmed diagnosis of infection, (2) shock (both by previous definitions [12]: SBP < 90 mmHg or MAP < 65 mmHg after fluid challenge or LA > 4 mmol/l; and by Sepsis-3 [18]: SBP < 90 mmHg or MAP < 65 mmHg and use of vasopressors and LA > 2 mmol/l), and (3) appropriate use of antimicrobials. In all these models, the crude effect of time of antimicrobial administration was estimated, as well as the effect adjusting for the propensity score and for confounding covariates such as age, Charlson Index, intravenous fluids, blood cultures in the first 3 h, lactate value, the SOFA, and the APACHE II scores. In addition, we analyzed the effect of each hour of delay in the administration of antimicrobials as a continuous independent variable for all previous regression models among the total study population. There was 2.2% of missing data, which were excluded for the generation of the propensity score. Statistical analyses were performed with the STATA version 14 Program (StataCorp, College Station, TX) and R version 3.4.1. The full paper and the data followed the STROBE statement [19].

## Results

### Baseline characteristics before propensity matching

**Fig. 1:**
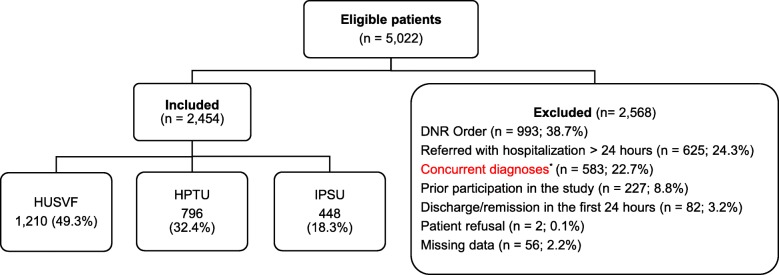
Flowchart for the study population. HUSVF, Hospital Universitario San Vicente Fundación; HPTU, Hospital Pablo Tobón Uribe; IPSU, IPS Universitaria León XIII; DNR, do-not-resuscitate order. ^*^Cirrhosis (*n* = 181; 31.1%); surgery < 24 h (*n* = 98; 16.8%); gastrointestinal bleeding (*n* = 89; 15.3%); CD4 count < 50 cells/mm3 (*n* = 75; 12.9%); diabetic ketoacidosis or hyperosmolar state (*n* = 57; 9.8%); seizures not due to meningitis (*n* = 35; 6.0%); myocardial infarction (*n* = 12; 2.1%), trauma (*n* = 11; 1.9%), asthmatic crisis (*n* = 9; 1.5%), pregnancy (*n* = 6; 1.0%), arrhythmia (*n* = 6; 1.0%), acute episode of cerebrovascular disease (*n* = 2; 0.3%), burns (*n* = 1; 0.2%), and thyrotoxicosis (*n* = 1; 0.2%)

A total of 5022 patients were screened, of which 2454 entered the study (Fig. 1). The clinical, physiological, and laboratory parameters of the pre- and post-matched population are presented according to the administration of antimicrobials within the first hour (Table 1) or 3 h (Table 2). The additional files 1 and 2 include all the variables taken into account for the PS model.

**Table 1:** Use of antimicrobials within the first hour of admission to the ED, with and without propensity score matching

Variable	Pre-match				Post-match			
	> 1 h, ***n*** = 2114 (86.2%)	< 1 h, ***n*** = 340 (13.8%)	***p*** value	SMD (%)	> 1 h, ***n*** = 340 (50%)	< 1 h, ***n*** = 340 (50%)	***p*** value	SMD (%)
Age	62 (48–75)	59 (42–70)	< 0.001	27.4	60 (43–71)	59 (42–70)	0.757	1.3
Systolic blood pressure	110 (90–131)	89 (80–115)	< 0.001	72.5	95 (82–116)	89 (80–115)	0.037	9.9
Temperature °C	37 (36.7–38.3)	37.8 (37–38.7)	< 0.001	34.7	37.5 (37–38.7)	37.8 (37–38.7)	0.462	6.9
Heart rate	103 (88–117)	110 (99–123)	< 0.001	43.7	110 (96–120)	110 (99–123)	0.274	9.9
Respiratory rate	19 (18–22)	21 (18–25)	< 0.001	38	20 (18–23)	21 (18–25)	0.027	12.9
Lactate	2.5 (1.5–3.5)	2.3 (1.3–3.4)	0.086	8.2	2.1 (1.2–3.2)	2.3 (1.3–3.4)	0.139	8.8
Central venous catheter	144 (6.8%)	37 (10.9%)	0.008	9.9	29 (8.5%)	37 (10.9%)	0.300	4.3
Fluids (IVF) in the first 6 h	1608 (76.1%)	313 (92.1%)	< 0.001	40.5	314 (92.4%)	313 (92.1%)	0.886	0.5
Amount of fluids in 6 h	955 (150–1500)	1500 (637–2325)	< 0.001	63.3	1450 (500–2150)	1500 (637–2325)	0.290	15.5
Urinary output	646 (30.6%)	171 (50.3%)	< 0.001	39.9	159 (46.8%)	171 (50.3%)	0.357	5.8
Vasopressors	299 (14.1%)	93 (27.4%)	< 0.001	28.2	84 (24.7%)	93 (27.4%)	0.432	4.5
Blood cultures	1819 (86.1%)	321 (94.4%)	< 0.001	21.9	324 (95.3%)	321 (94.4%)	0.603	1.7
Blood cultures taken prior to the beginning of antibiotics	1508 (71.3%)	129 (37.9%)	< 0.001	68.5	138 (40.6%)	129 (37.9%)	0.480	4.4
Admission to ICU/SCU	856 (40.5%)	182 (53.5%)	< 0.001	26.4	179 (52.7%)	182 (53.5%)	0.818	1.4
Mechanical ventilation	311 (14.7%)	65 (19.1%)	0.036	9.9	63 (18.5%)	65 (19.1%)	0.844	1
Confirmed diagnosis of infection	1624 (76.8%)	285 (83.8%)	0.004	16.2	263 (77.4%)	285 (83.8%)	0.033	11.5
Inadequate antimicrobials	610 (28.9%)	42 (12.4%)	< 0.001	39.6	45 (13.2%)	42 (12.4%)	0.731	1.6
Mortality*	240 (11.4%)	43 (12.7%)	0.488	3.1	40 (11.8%)	43 (12.7%)	0.725	1.6
Hospital stay	10 (6–16)	11 (6–19)	0.002	21.2	11 (6–19)	11 (6–19)	0.335	2.3
SOFA score*	4 (2–6)	5 (3–7)	< 0.001	43.2	4 (3–7)	5 (3–7)	0.473	9.2
APACHE II score*	14 (9–12)	15 (11–19)	< 0.001	32.4	15 (9–18)	15 (11–19)	0.148	14.0
Charlson Index*	1 (0–2)	1 (0–2)	0.010	18.2	1 (0–2)	1 (0–2)	0.430	2.2
h between admission and administration of antibiotics*	6 (4–10)	1 (1–1)	< 0.001	-	4 (2–8)	1 (1–1)	< 0.001	-

The measurements for continuous variables are the median (IQR) and for categorical: *n* (%). *SMD* standardized mean difference

*The variables were not included to generate the propensity score

**Table 2:** Use of antimicrobials within the 3 h of admission to the ED, with and without propensity score matching

Variable	Pre-matching				Post-matching			
	> 3 h, ***n*** = 1673 (68.2%)	< 3 h, ***n*** = 781 (31.8%)	***p*** value	SMD (%)	> 3 h, ***n*** = 781 (50%)	< 3 h, ***n*** = 781 (50%)	***p*** value	SMD (%)
Age	63 (47–75)	60 (45–72)	0.004	15.9	61 (45–73)	60 (45–72)	0.541	3
Systolic blood pressure	115 (93–134)	93 (80–119)	< 0.001	73.6	100 (85–124)	93 (80–119)	< 0.001	20.1
Temperature °C	37 (36.7–38.3)	37.3 (37–38.5)	< 0.001	17.6	37.2 (36.9–38.5)	37.3 (37–38.5)	0.318	4.6
Heart rate	102 (88–116)	110 (95–120)	< 0.001	38.2	106 (90–120)	110 (95–120)	0.123	10.1
Respiratory rate	19 (18–22)	20 (18–25)	< 0.001	39.3	20 (18–24)	20 (18–25)	0.002	10.6
Lactate	2.6 (1.6–3.5)	2.3 (1.3–3.4)	< 0.001	1.9	2.4 (1.3–3.6)	2.3 (1.3–3.4)	0.354	0.5
Central venous catheter	88 (5.3%)	93 (11.9%)	< 0.001	12.8	71 (9.1%)	93 (11.9%)	0.069	5.1
Fluids (IVF) in the first 6 h	1212 (72.4%)	709 (90.8%)	< 0.001	36	704 (90.1%)	709 (90.8%)	0.667	1.2
Amount of fluids in 6 h	660 (0–1500)	1420 (550–2300)	< 0.001	70.6	1210 (500–2000)	1420 (550–2300)	0.006	21.3
Urinary output	451 (27%)	366 (46.9%)	< 0.001	34.1	306 (39.2%)	366 (46.9%)	0.002	12.5
Vasopressors	190 (11.4%)	202 (26.9%)	< 0.001	25.9	156 (20%)	202 (25.9%)	0.006	10
Blood cultures	1411 (84.3%)	729 (93.3%)	< 0.001	18.1	714 (91.4%)	729 (93.3%)	0.153	3.6
Blood cultures taken prior to the beginning of antibiotics	1216 (72.7%)	421 (53.9%)	< 0.001	32.1	535 (68.5%)	421 (53.9%)	< 0.001	23.8
Admission to ICU/SCU	628 (37.5%)	410 (52.5%)	< 0.001	25.6	377 (48.3%)	410 (52.5%)	0.095	6.9
Mechanical ventilation	217 (13%)	159 (20.4%)	< 0.001	13.5	149 (19.1%)	159 (20.4%)	0.525	2.2
Confirmed diagnosis of infection	1266 (75.7%)	643 (82.3%)	< 0.001	12.4	617 (79%)	643 (82.3%)	0.096	5.9
Inadequate antimicrobials	527 (31.5%)	125 (16%)	< 0.001	29.1	149 (19.1%)	125 (16%)	0.110	5.5
Mortality*	179 (10.7%)	104 (13.3%)	0.059	5	105 (13.4%)	104 (13.3%)	0.941	0.2
Hospital stay	10 (6–16)	11 (6–18)	< 0.001	21.2	10 (6–17)	11 (6–18)	0.153	7.5
SOFA score*	3 (2–5)	4 (3–7)	< 0.001	48.5	4 (3–6)	4 (3–7)	0.034	17.7
APACHE II score*	13 (9–18)	15 (10–19)	< 0.001	31.5	14 (10–19)	15 (10–19)	0.052	13.2
Charlson Index*	1 (0–2)	1 (0–2)	0.010	16.2	1 (0–2)	1 (0–2)	0.101	6.4

The measurements for continuous variables are the median (IQR) and for categorical: *n* (%). *SMD* standardized mean difference

*The variables were not included to generate the propensity score

The median age of the patients was 62 years (IQR = 46–74) and 50% (*n* = 1227) were women. The most frequent diagnoses at admission were urinary tract infection (UTI) and pneumonia with 27.8% (*n* = 682) and 27.3% (*n* = 671), respectively. Altogether, 2140 (87%) of the patients had blood cultures, of which 29.4% (*n* = 629) were positive; the most frequent microorganisms were *Escherichia coli* (36.6%; *n* = 230), *Staphylococcus aureus* (21%; *n* = 132), and *Klebsiella pneumoniae* (10.2%; *n* = 64). A 76.5% (*n* = 1637) of the blood cultures were taken prior to the start of the antimicrobial, 90.3% (*n* = 2215) of the patients received antimicrobials in the first 24 h and started at a median of 5 h from the time of arrival at the ED (IQR = 2–9), 31.8% (*n* = 781) in the first 3 h and 13.9% (*n* = 340) in the first hour of admission to the ED. The most commonly used antimicrobials were piperacillin/tazobactam in 53.7% (*n* = 1189), ampicillin/sulbactam in 10.1% (*n* = 223), and meropenem in 6.6% (*n* = 145). The diagnosis of infection was confirmed in 1909 patients (77.8%) and among them, 23.5% (*n* = 449) had an inadequate prescription, according to microbiological or clinical criteria. Some clinical care variables in the first hour of administration of antimicrobials were central venous catheter placement (10.9% vs. 6.8%, *p* = 0.008), red blood cell transfusion (4.1% vs. 1.8%, *p* = 0.007), onset of vasopressors in the first 24 h (27.4% vs. 14.1%, *p* = <0.001), and request of blood cultures (94.4% vs. 86.1%, *p* = <0.001). Among the total cohort, 42.3% of the patients (*n* = 1038) were admitted to the intensive care unit (ICU) and 15.3% (*n* = 376) required mechanical ventilation. When applying definitions for the identification of patients with septic shock, it was found that 35.4% (*n* = 869) and 7.3% (*n* = 179) met the older and newest diagnostic criteria, respectively. The total cohort in-hospital mortality rate was 11.5% (*n* = 283); divided by antimicrobial administration time, it was < 1 h 12.7% (*n* = 43), < 3 h 13.3% (*n* = 104), and > 3 h 10.7% (*n* = 179).

The SMD was much larger than 10% in most of the variables analyzed in the unmatched population, both within the first hour or within 3 h (Tables 1 and 2). The variables SBP, amount of fluids in the first 6 h, urinary output, use of vasopressors, and admission to ICU, seems to indicate that the population that received the antimicrobial earlier was more critically ill.

### Baseline characteristics after propensity matching

After matching for the propensity score, a population of 340 patients with antimicrobials within the first hour and 340 with subsequent antimicrobial treatment was obtained (Table 1). The same pairing procedure by propensity score obtained a population of 781 patients with antimicrobials within the first 3 h and 781 with subsequent antimicrobial treatment (Table 2).

For the first hour comparison, SMD was < 10% for most variables after PS matching, except respiratory rate (12.9%), creatinine (11.9%), amount of fluids in the first 6 h (15.5%), and confirmed diagnosis of infection (11.5%). On the other hand, for the first 3 h, the variables with SMD > 10% after matching by PS were systolic blood pressure (20.1%), amount of fluids in the first 6 h (21.3%), urinary output (12.5%), blood cultures taken prior to the beginning of antibiotics (23.8%), and site of infection (10.7%).

### Outcome models

#### General model

For the matched population, in the general model adjusting for propensity score and confounding covariates, there were no significant differences in mortality with antimicrobial administration within or after the first hour (≤ 1 h vs > 1 h) (OR 1.03; 95% CI = 0.63; 1.70) nor within the first 3 h or after (≤ 3 h vs > 3 h) (OR 0.85; 95% CI = 0.61; 1.20) (Table 3).

**Table 3:** Effect of the antimicrobial administration either within 1 or 3 hours of admission to the ED, matched by PS, on in-hospital mortality

	Antimicrobials ≤ 1 h vs. > 1 h, OR (95% CI)	Antimicrobials ≤ 3 h vs. > 3 h, OR (95% CI)
Models	*n* = 680	*n* = 1562
No adjustment	1.09 (0.69; 1.72)	0.98 (0.74; 1.32)
Adjusted for propensity score	1.05 (0.66; 1.67)	0.86 (0.63; 1.17)
Adjusted for covariates*	1.07 (0.65; 1.75)	0.93 (0.67; 1.28)
Adjusted for propensity score + covariates *	1.03 (0.63; 1.70)	0.85 (0.61; 1.20)
Patients with confirmed infection	*n* = 548	*n* = 1260
No adjustment	1.00 (0.61; 1.64)	1.00 (0.73; 1.38)
Adjusted for propensity score	0.99 (0.61; 1.63)	0.89 (0.64; 1.25)
Adjusted for covariates**	0.92 (0.54; 1.58)	0.91 (0.64; 1.30)
Adjusted for propensity score + covariates**	0.90 (0.53; 1.56)	0.88 (0.61; 1.26)
Patients with adequate antibiotics	*n* = 593	*n* = 1288
No adjustment	0.96 (0.59; 1.56)	0.98 (0.71; 1.36)
Adjusted for propensity score	0.93 (0.57; 1.53)	0.82 (0.58; 1.16)
Adjusted for covariates***	0.88 (0.51; 1.50)	0.89 (0.62; 1.27)
Adjusted for propensity score + covariates***	0.86 (0.50; 1.46)	0.82 (0.56; 1.19)
Patients with shock—Rivers	*n* = 342	*n* = 702
No adjustment	0.96 (0.54; 1.73)	0.98 (0.66; 1.44)
Adjusted for propensity score	0.91 (0.50; 1.66)	0.87 (0.57; 1.32)
Adjusted for covariates*	0.92 (0.49; 1.74)	0.92 (0.59; 1.42)
Adjusted for propensity score + covariates*	0.87 (0.46; 1.66)	0.85 (0.54; 1.35)
Patients with shock—Sepsis-3	*n* = 91	*n* = 170
No adjustment	0.85 (0.34; 2.15)	0.74 (0.39; 1.43)
Adjusted for propensity score	0.70 (0.26; 1.84)	0.56 (0.27; 1.15)
Adjusted for covariates*	0.86 (0.31; 2.37)	0.86 (0.40; 1.85)
Adjusted for propensity score + covariates*	0.75 (0.26; 2.14)	0.65 (0.28; 1.51)

^*^Covariates: age, Charlson Index, intravenous fluids ≥ 1500 first hour, blood cultures in the first 3 h, lactate, SOFA score, APACHE II score, confirmed diagnosis of infection, and inadequate antimicrobials

^**^Covariates: age, Charlson Index, intravenous fluids ≥ 1500 first hour, blood cultures in the first 3 h, lactate, SOFA score, APACHE II score, and inadequate antimicrobials

^***^Covariates: age, Charlson Index, intravenous fluids ≥ 1500 first hour, blood cultures in the first 3 h, lactate, SOFA score, APACHE II score, and confirmed diagnosis of infection

#### Subgroups models

Similar results were found when analyzing the population with confirmed infection, adequate use of antibiotics, and septic shock according to two different definitions (Table 3). In addition, it was not possible to demonstrate a significant increase in-hospital mortality for each hour of delay in the administration of antimicrobials, nor in the crude analysis (OR = 0.98; 95% CI = 0.96; 1.01), nor in the analysis adjusted for covariates (OR = 1.00; 95% CI = 0.97; 1.03) (Table 4).

**Table 4:** Effect of each hour of delay in the administration of antimicrobials since admission to the ED on in-hospital mortality

Models	Type of logistic regression	
	No adjustment	Adjusted for covariates
	OR (95% CI)	OR (95% CI)
Models with total population (*n* = 2215)	0.98 (0.96; 1.01)	1.00^*^ (0.97; 1.03)
Models with confirmed infection (*n* = 1751)	0.98 (0.96; 1.01)	1.00^**^ (0.97; 1.03)
Models with not confirmed infection (*n* = 464)	1.00 (0.94; 1.07)	1.03^**^ (0.95; 1.10)
Models with adequate antibiotics (*n* = 1802)	0.98 (0.95; 1.01)	1.01^***^ (0.97; 1.03)
Models with not adequate antibiotics (*n* = 413)	0.99 (0.94; 1.05)	1.01^***^ (0.95; 1.07)
Models with shock—Rivers (*n* = 830)	0.99 (0.96; 1.03)	1.01^*^ (0.96; 1.05)
Models with shock—Sepsis-3 (*n* = 179)	1.06 (0.98; 1.14)	1.03^*^ (0.94; 1.12)

^*^Covariates: age, Charlson Index, intravenous fluids ≥ 1500 first hour, blood cultures in the first 3 h, lactate, SOFA score, APACHE II score, confirmed diagnosis of infection, and inadequate antimicrobials

^**^Covariates: age, Charlson Index, intravenous fluids ≥ 1500 first hour, blood cultures in the first 3 h, lactate, SOFA score, APACHE II score, and inadequate antimicrobials

^***^Covariates: age, Charlson Index, intravenous fluids ≥ 1500 first hour, blood cultures in the first 3 h, lactate, SOFA score, APACHE II score, and confirmed diagnosis of infection

#### Total population

When analyzing the total unmatched population, none of these different models showed significant differences in mortality with the administration of antimicrobials within 1 h or 3 h (Additional file 3).

## Discussion

In this secondary analysis of a multicenter prospective cohort of patients admitted to the emergency department with suspected sepsis, septic shock, or infection with organ dysfunction, the early administration of antimicrobials was not associated with a significant decrease in in-hospital mortality when comparing different time cut offs (≤ 1 h vs > 1 h nor ≤ 3 h vs > 3 h). Nor was an association found between each hour of delay in the administration of antimicrobials and an increase in mortality.

These results appear to be contrary to current recommendations, in which the administration of appropriate antibiotics within the first hour of recognition of sepsis is considered a standard of management quality [20]. The foregoing has been based on several observational studies that indicate that the delay in antibiotic administration is associated with higher mortality [21–24]. A retrospective study of a large administrative database that included 35,000 patients with sepsis, severe sepsis, and septic shock concluded that every hour of delay in the administration of antibiotics is associated with a higher probability of hospital mortality adjusted for the characteristics of the patient and the severity of the disease (OR, 1.09; 95% CI, 1.05–1.13); although, it is important to consider that in this study, no data were presented on confirmation of infection nor adequacy of antimicrobials [21]. In the study by Seymour et al., of 49,331 patients with severe sepsis or septic shock, an association was also found between the time elapsed in the administration of antibiotics and hospital mortality (OR, 1.04 per hour; 95% CI, 1.03–1.06) [22]. Reinforcing these findings, in a retrospective cohort with 5072 patients, Pruinelli et al. found that a 125-min delay in the onset of antibiotics increases the risk of mortality, concluding that very short delays could have an adverse impact on outcomes [25]. A recent systematic review concluded that the benefit of antibiotic use within the first hour was greater in patients with septic shock [26]. Additionally, supporting the benefit of early antimicrobials in those most critically ill patients, in our primary analysis that included 884 adult patients admitted to the ED with infection and shock according to the previous definition (systolic blood pressure < 90 mmHg after fluids challenge or lactate > 4 mmol/L), we found a potential absolute reduction in mortality of 21% with the use of antibiotics in the first 3 h [10]. However, in that analysis, we used an instrumental variable approach for comparison among all of the components of the full EGDT protocol, trying to establish the weight of each one in the outcomes of patients.

Although the usefulness of antimicrobials is not in doubt, the controversy persists as to whether the evidence is strong enough to support current early administration guidelines. In this regard, it is stated that the evidence for “every hour of delay” is based solely on retrospective analyses of administrative databases and it is emphasized that these designs are missing crucial data, such as the confirmation of infection, the appropriate choice of antimicrobials with their doses, and control of the source of infection, important confounding factors with possible influence on outcomes [27]. In a prospective and multicenter study by de Groot et al, 1168 patients were stratified in three categories of severity according to the predisposition, infection, response, and organ failure score (PIRO, score 1 to 7, 8 to 14 and > 14 points), without finding a reduction in mortality according to the time of onset of the antibiotics in any of the categories [28]. In a before-and-after study conducted in patients with suspected infection in a surgical intensive care unit (ICU), Hranjec et al. compared an aggressive regimen of antibiotic administration (initiation of antibiotics in all patients with suspected infection) with a conservative regimen (starting only after objective confirmation of infection), finding that the former was associated with higher mortality (OR, 2.5; 95% CI, 1.5–4.0) [7]. In addition, in a prospective observational study that enrolled 1184 adult patients diagnosed with severe sepsis and septic shock, Abe et al. were unable to show a linear relationship between the timing of antibiotic administration, such as within 1 h or 3 h after sepsis recognition, and in-hospital mortality, nor a relation between time to antibiotics as a continuous variable and mortality (OR, 0.99; 95% CI 0.99–1.0; *p* = 0.152) [29]. In our knowledge, the only randomized controlled trial (RCT) that evaluated early antimicrobials administration in 2672 patients with suspected infection, although in a prehospital setting, failed to find an association between early administration of antimicrobials and mortality (RR = 0.95; 95% CI, 0.74–1.24) [30].

Additionally, our results also agree with the conclusions of the systematic review by Sterling et al., who evaluated two scenarios. In the first scenario, the authors compared patients who received antibiotics 3 or more hours after admission to the emergency room with those who received antibiotics within the first 3 h, without finding a significant increase in mortality (OR, 1.16; 95% CI, 0.92–1.46). For the second scenario, they compared the administration after 1 h of recognizing of the shock with the administration in the first hour, also indicating that early administration is not associated with a significant benefit in mortality (OR, 1.46; 95% CI, 0.89–2.40) [6]. In this regard, it should be noted that in our study, when doing a subgroup analysis, we did not find significant differences in mortality with the administration of antimicrobials within 1 h or within 3 h of admission to the ED in patients with septic shock, in contrast to emerging literature that suggests a greater benefit in this population [21, 22, 26]. These findings are similar to the results of a recent small observational study that included 150 septic shock patients admitted to the medical intensive care unit (MICU) with an in-hospital mortality rate of 49.3%; this study did not show an association between timing of antibiotic administration and mortality [31]. All this would suggest a more prudent—albeit critical—approach to the consideration of the timely and adequate initiation of antimicrobials, in which the initial resuscitation measures and the determination of the focus of infection would be critical.

Among the strengths of our study is its character as a multicenter prospective cohort, and not based on an administrative database, with a fairly representative population of the heterogeneous clinical presentation of sepsis in the ED, in addition to a reliable collection of crucial data about the confirmation of infection, the administration time of the antimicrobials, and the adequacy of their prescription, based on the resistance profile of the microorganisms. In the analysis, in addition to the exhaustive adjustment for various covariates, novel statistical techniques like the PS were considered to try to achieve a balance of the groups and estimate more accurately the effect of the intervention, given the logistical and ethical difficulty to perform a RCT in the field.

### Limitations

It is necessary to highlight the restriction regarding the sample size, for which it is not possible to rule out a beneficial effect, although it is important to note that our sample size is one of the largest collected prospectively in a study that shows no association between early administration of antimicrobials and mortality in patients with sepsis [7, 28–32]. Also, the problem of residual confounding—despite the use of the propensity score and adjustment for covariates—cannot be eliminated. With regard to the definitions of antimicrobial administration time (time zero), our reference to the delay in administration was from admission to the ED, which we consider more relevant from the clinical point of view, while in some previous research it is conceived from the onset of hypotension or recognition of shock [33]. In addition, in our study, we only evaluate in-hospital mortality, so we cannot conclude anything about the long-term effects, a topic analyzed in other research [34, 35]. Another consideration is that, during the recruitment period of the study population, the Sepsis-3 criteria were not available. However, in the current study, we analyzed the eligible population with any organ dysfunction, similar to the current process based on the SOFA score. Indeed, the median SOFA score in our cohort was 4 or 5 for antibiotics > or < 1 h, respectively.

The overall mortality rate in our study was lower (11.5%), compared to previous, retrospective (56%) [33] and prospective (19%) [32] studies, which may decrease the power to detect differences; the relatively high rates of UTI (27.8%) compared to other studies might account for this lower rate. Nevertheless, it is important to highlight that previous studies had a lower mortality rate in patients with diagnosis of sepsis (3.3%) and severe sepsis (8.8%) [21]. In addition, in our study, only ~ 50% of patients who received antimicrobials within the first hour were admitted to the ICU and about 25% received vasopressors. Thus, it appears that the present cohort had a lower severity of illness than previous studies, as well as a lower proportion of patients with septic shock, population in which the literature seems to suggest a greater benefit from the early administration of antimicrobials [10, 21, 22, 26].

### Interpretation and implications

The benefit of early administration in this last population perhaps was not reflected in our study due to the aforementioned limitations, so a cautious interpretation of these findings is necessary, since we do not suggest delaying the use of antimicrobials in patients with septic shock despite the current moderate evidence. However, another is the scenario for patients with sepsis and suspected sepsis, a population that is represented in our study in the daily context of an ED and which has also been matched by multiple covariates and confounding factors. In this population without shock, the association between the early administration of antimicrobials and mortality has been null or minimal, a situation also evidenced in our study [21, 22, 30]. If we take this into account, the most recent recommendations, in which the rapid and aggressive use of antimicrobials is done even in patients with no infection to simply meet a benchmark, would not be justified in this context, where some clinical important information could be collected to ensure the presence of infection without increased risk of adverse outcomes [36].

## Conclusion

In the present study, it was not possible to demonstrate a statistically significant association between the early administration of antimicrobials and mortality in sepsis patients with or without shock. However, according to the wide confidence interval, we cannot discard a beneficial effect of earlier antibiotics in patients with septic shock. These findings reinforce the idea that more research is needed in the field and suggest a more comprehensive approach to sepsis and its treatment, considering early detection, multiple interventions, and goals beyond the simple time of antimicrobial administration, which may favor the indiscriminate use of them.

## Supplementary information

**Additional file 1.** Use of antimicrobials within the first hour of admission to the ED, with and without propensity score matching.

**Additional file 2.** Use of antimicrobials within the three hours of admission to the ED, with and without propensity score matching.

**Additional file 3.** Effect of the antimicrobial administration in the first hours of admission to the ED on in-hospital mortality. Models with total population.

## Data Availability

The datasets analyzed are available with the corresponding author on reasonable request.

## Abbreviations

ED: Emergency department
IQR: Interquartile range
OR: Odds ratio
HUSVF: Hospital Universitario San Vicente Fundación
HPTU: Hospital Pablo Tobón Uribe
IPSU: IPS Universitaria León XIII
CDC: Center for Disease Control and Prevention
CR: Creatinine
INR: International normalized ratio
PTT: Partial thromboplastin time
PLT: Platelet count
LA: Lactic acid
SBP: Systolic blood pressure
MAP: Mean arterial pressure
PS: Propensity score
SMD: Standardized mean difference
ICU: Intensive care unit
