# Supplementary material for: Antimicrobials administration time in patients with suspected sepsis: is faster better? An analysis by propensity score
Source: J Intensive Care. 2020 Apr 22;8:28. doi: 10.1186/s40560-020-00448-1 (PMC7178597; doi:10.1186/s40560-020-00448-1)
Supplement: Supplementary file 1 — Additional file 1. Use of antimicrobials within the first hour of admission to the ED, with and without propensity score matching. [file 40560_2020_448_MOESM1_ESM.docx]

**Additional file 1. Use of antimicrobials within the first hour of admission to the ED, with and without propensity score matching**

| Variable | Pre-match | | | | | Post-match | | | |
| --- | --- | --- | --- | --- | --- | --- | --- | --- | --- |
|  | > 1 hour  n=2,114 (86.2%) | < 1 hour  n=340 (13.8%) | p value | SMD (%) | > 1 hour  n=340 (50%) | | < 1 hour  n=340 (50%) | p value | SMD (%) |
| Age | 62 (48 – 75) | 59 (42 – 70) | <0.001 | 27.4 | 60 (43 – 71) | | 59 (42 – 70) | 0.7568 | 1.3 |
| Hospital on admission | | | | | | | | | |
| HUSVF  HPTU  IPSU | 1099 (52%)  421 (19.9%)  594 (28.1%) | 111 (32.7%)  27 (7.9%)  202 (59.4%) | <0.001 | 68.6 | 104 (30.6%)  66 (19.4%)  170 (50%) | | 111 (32.7%)  27 (7.9%)  202 (59.4%) | <0.001 | 8.9 |
| Systolic Blood Pressure | 110 (90 – 131) | 89 (80 – 115) | <0.001 | 72.5 | 95 (82 – 116) | | 89 (80 – 115) | 0.0372 | 9.9 |
| Diastolic Blood Pressure | 67 (52 – 80) | 56 (45 – 67) | <0.001 | 65.7 | 57 (49 – 70) | | 56 (45 – 67) | 0.0610 | 10.9 |
| PaO_2_/FiO_2_  Index | 295 (222 – 367) | 284 (196 – 360) | 0.0453 | 12.5 | 291 (210 – 372) | | 284 (196 – 360) | 0.2801 | 10.4 |
| Platelets | 234000 (163000 – 322000) | 225000 (162000 – 303000) | 0.0962 | 10 | 226000 (162500 – 315500) | | 225000 (162000 – 303000) | 0.6390 | 0.7 |
| Temperature °C | 37 (36.7 – 38.3) | 37.8 (37 – 38.7) | <0.001 | 34.7 | 37.5 (37 – 38.7) | | 37.8 (37 – 38.7) | 0.4618 | 6.9 |
| Heart Rate | 103 (88 – 117) | 110 (99 – 123) | <0.001 | 43.7 | 110 (96 – 120) | | 110 (99 – 123) | 0.2741 | 9.9 |
| Respiratory rate | 19 (18 – 22) | 21 (18 – 25) | <0.001 | 38 | 20 (18 – 23) | | 21 (18 – 25) | 0.0271 | 12.9 |
| Serum potassium | 4.1 (3.7 – 4.6) | 4 (3.6 – 4.5) | 0.0399 | 11.4 | 4.1 (3.6 – 4.5) | | 4 (3.6 – 4.5) | 0.4314 | 4.5 |
| Hematocrit | 37.2 (32.3 – 42.1) | 36.4 (30 – 41.6) | 0.0615 | 14.5 | 37 (30 – 42) | | 36.4 (30 – 42) | 0.7264 | 3.8 |
| White blood cells | 13200 (8800 – 18300) | 12400 (8900 – 18450) | 0.2885 | 12.4 | 12670 (7905 – 17100) | | 12400 (8900 – 18450) | 0.4790 | 7.2 |
| Creatinine | 1.2 (0.8 – 2.3) | 1.3 (0.8 – 2.3) | 0.9404 | 5.7 | 1.3 (0.8 – 2.3) | | 1.3 (0.8 – 2.3) | 0.8034 | 11.9 |
| Lactate | 2.5 (1.5 – 3.5) | 2.3 (1.3 – 3.4) | 0.0855 | 8.2 | 2.1 (1.2 – 3.2) | | 2.3 (1.3 – 3.4) | 0.1387 | 8.8 |
| Central venous catheter | 144 (6.8%) | 37 (10.9%) | 0.008 | 9.9 | 29 (8.5%) | | 37 (10.9%) | 0.300 | 4.3 |
| Fluids (IVF) in the first six hours | 1608 (76.1%) | 313 (92.1%) | <0.001 | 40.5 | 314 (92.4%) | | 313 (92.1%) | 0.886 | 0.5 |
| Amount of fluids in six hours | 955 (150 – 1500) | 1500 (637 – 2325) | <0.001 | 63.3 | 1450 (500 – 2150) | | 1500 (637 – 2325) | 0.2898 | 15.5 |
| Urinary Output | 646 (30.6%) | 171 (50.3%) | <0.001 | 39.9 | 159 (46.8%) | | 171 (50.3%) | 0.357 | 5.8 |
| Transfusions | 39 (1.8%) | 14 (4.1%) | 0.007 | 6.1 | 11 (3.2%) | | 14 (4.1%) | 0.541 | 1.7 |
| Vasopressors | 299 (14.1%) | 93 (27.4%) | <0.001 | 28.2 | 84 (24.7%) | | 93 (27.4%) | 0.432 | 4.5 |
| Blood cultures | 1819 (86.1%) | 321 (94.4%) | <0.001 | 21.9 | 324 (95.3%) | | 321 (94.4%) | 0.603 | 1.7 |
| Blood cultures taken prior to the beginning of antibiotics | 1508 (71.3%) | 129 (37.9%) | <0.001 | 68.5 | 138 (40.6%) | | 129 (37.9%) | 0.480 | 4.4 |
| Admission to ICU/SCU | 856 (40.5%) | 182 (53.5%) | <0.001 | 26.4 | 179 (52.7%) | | 182 (53.5%) | 0.818 | 1.4 |
| Mechanical ventilation | 311 (14.7%) | 65 (19.1%) | 0.036 | 9.9 | 63 (18.5%) | | 65 (19.1%) | 0.844 | 1 |
| Site of Infection | | | | | | | | | |
| Urinary Tract Infection | 602 (28.5%) | 80 (23.5%) | 0.012 | 10.9 | 97 (28.5%) | | 103 (30.3%) | 0.261 | 3 |
| Pneumonia | 568 (26.9%) | 103 (30.3%) |  |  | 88 (25.9%) | | 103 (30.3%) |  |  |
| Intra-abdominal infection | 218 (10.3%) | 50 (14.7%) |  |  | 45 (13.2%) | | 50 (14.7%) |  |  |
| Skin and soft tissues | 208 (9.8%) | 21 (6.2%) |  |  | 23 (6.8%) | | 21 (6.2%) |  |  |
| Blood stream | 153 (7.2%) | 33 (9.7%) |  |  | 24 (7.1%) | | 33 (9.7%) |  |  |
| Unknown source | 135 (6.4%) | 22 (6.4%) |  |  | 35 (10.3%) | | 22 (6.5%) |  |  |
| Others | 230 (10.9%) | 31 (9.1%) |  |  | 28 (8.2%) | | 31 (9.1%) |  |  |
| Confirmed diagnosis of infection | 1624 (76.8%) | 285 (83.8%) | 0.004 | 16.2 | 263 (77.4%) | | 285 (83.8%) | 0.033 | 11.5 |
| Inadequate antimicrobials | 610 (28.9%) | 42 (12.4%) | <0.001 | 39.6 | 45 (13.2%) | | 42 (12.4%) | 0.731 | 1.6 |
| Mortality * | 240 (11.4%) | 43 (12.7%) | 0.488 | 3.1 | 40 (11.8%) | | 43 (12.7%) | 0.725 | 1.6 |
| Hospital stay | 10 (6 – 16) | 11 (6 – 19) | 0.0023 | 21.2 | 11 (6 – 19) | | 11 (6 – 19) | 0.3347 | 2.3 |

The measurements for continuous variables are the median (IQR) and for categorical: n (%). SMD: standardized mean difference

***** The Mortality variable was not included to generate the propensity score.
