# Supplementary material for: Antimicrobials administration time in patients with suspected sepsis: is faster better? An analysis by propensity score
Source: J Intensive Care. 2020 Apr 22;8:28. doi: 10.1186/s40560-020-00448-1 (PMC7178597; doi:10.1186/s40560-020-00448-1)
Supplement: Supplementary file 2 — Additional file 2. Use of antimicrobials within the three hours of admission to the ED, with and without propensity score matching. [file 40560_2020_448_MOESM2_ESM.docx]

**Additional file 2. Use of antimicrobials within the three hours of admission to the ED, with and without propensity score matching**

| Variable | Pre-matching | | | | Post-matching | | | |
| --- | --- | --- | --- | --- | --- | --- | --- | --- |
|  | > 3 hours  n = 1,673 (68.2%) | <3 hours  n = 781 (31.8%) | p value | SMD (%) | > 3 hours  n=781  (50%) | < 3 hours  n=781  (50%) | p value | SMD (%) |
| Age | 63 (47 – 75) | 60 (45  – 72) | 0.0040 | 15.9 | 61 (45 – 73) | 60 (45 – 72) | 0.5414 | 3 |
| Hospital on admission | | | | | | | | |
| HUSVF  HPTU  IPSU | 951 (56.8%)  324 (19.4%)  398 (23.8%) | 259 (33.2%)  124 (15.9%)  398 (50.9%) | <0.001 | 64.5 | 314 (40.2%)  170 (21.8%)  297 (38%) | 259 (33.2%)  124 (15.9%)  398 (51%) | <0.001 | 24.7 |
| Systolic Blood Pressure | 115 (93  – 134) | 93 (80  – 119) | <0.001 | 73.6 | 100 (85  – 124) | 93 (80 – 119) | <0.001 | 20.1 |
| Diastolic Blood Pressure | 70 (55 – 80) | 57 (48 – 70) | <0.001 | 72.3 | 60 (49 – 75) | 57 (48 – 70) | 0.0076 | 18.2 |
| PaO_2_/FiO_2_  Index | 298 (228  – 370) | 281 (200 – 357) | <0.001 | 25.6 | 286 (214  – 361) | 281 (200  – 357) | 0.1929 | 10.3 |
| Platelets | 236000  (165000  – 324000) | 228000 (162000  – 307000) | 0.0301 | 15.2 | 232000  (161000  – 315000) | 228000  (162000 – 307000) | 0.3968 | 7.3 |
| Temperature °C | 37 (36.7 – 38.3) | 37.3 (37 – 38.5) | <0.001 | 17.6 | 37.2 (36.9 – 38.5) | 37.3 (37 – 38.5) | 0.3184 | 4.6 |
| Heart rate | 102 (88  – 116) | 110 (95  – 120) | <0.001 | 38.2 | 106 (90  – 120) | 110 (95  – 120) | 0.1234 | 10.1 |
| Respiratory rate | 19 (18  – 22) | 20 (18  – 25) | <0.001 | 39.3 | 20 (18  – 24) | 20 (18  – 25) | 0.0023 | 10.6 |
| Serum potassium | 4.1 (3.7 – 4.6) | 4 (3.6 – 4.5) | <0.001 | 14 | 4 (3.6 – 4.5) | 4 (3.6 – 4.5) | 0.3587 | 4.4 |
| Hematocrit | 37.3 (32.7 – 42.1) | 37 (31 – 42) | 0.0172 | 15.1 | 36.6 (31 – 41.8) | 37 (31 – 41.8) | 0.8084 | 4.1 |
| White blood cells | 13300 (9000 – 18300) | 12700 (8400 – 18500) | 0.0488 | 16.1 | 12800 (8200 – 18000) | 12700 (8400 – 18400) | 0.7489 | 5.4 |
| Creatinine | 1.2 (0.8  – 2.3) | 1.3 (0.8  – 2.2) | 0.7208 | 6.7 | 1.3 (0.8  – 2.3) | 1.3 (0.8  – 2.2) | 0.6806 | 3.7 |
| Lactate | 2.6 (1.6  – 3.5) | 2.3 (1.3  – 3.4) | <0.001 | 1.9 | 2.4 (1.3  – 3.6) | 2.3 (1.3  – 3.4) | 0.3535 | 0.5 |
| Central venous catheter | 88 (5.3%) | 93 (11.9%) | <0.001 | 12.8 | 71 (9.1%) | 93 (11.9%) | 0.069 | 5.1 |
| Fluids (IVF) in the first six hours | 1212 (72.4%) | 709 (90.8%) | <0.001 | 36 | 704 (90.1%) | 709 (90.8%) | 0.667 | 1.2 |
| Amount of fluids in six hours | 660 (0  – 1500) | 1420  (550  – 2300) | <0.001 | 70.6 | 1210  (500  – 2000) | 1420  (550  – 2300) | 0.0058 | 21.3 |
| Urinary Output | 451 (27%) | 366 (46.9%) | <0.001 | 34.1 | 306 (39.2%) | 366 (46.9%) | 0.002 | 12.5 |
| Transfusions | 31 (1.9%) | 22 (2.8%) | 0.126 | 2 | 17 (2.2%) | 22 (2.8%) | 0.417 | 1.2 |
| Vasopressors | 190 (11.4%) | 202 (26.9%) | <0.001 | 25.9 | 156 (20%) | 202 (25.9%) | 0.006 | 10 |
| Blood cultures | 1411 (84.3%) | 729 (93.3%) | <0.001 | 18.1 | 714 (91.4%) | 729 (93.3%) | 0.153 | 3.6 |
| Blood cultures taken prior to the beginning of antibiotics | 1216 (72.7%) | 421 (53.9%) | <0.001 | 32.1 | 535 (68.5%) | 421 (53.9%) | <0.001 | 23.8 |
| Admission to ICU/SCU | 628 (37.5%) | 410 (52.5%) | <0.001 | 25.6 | 377 (48.3%) | 410 (52.5%) | 0.095 | 6.9 |
| Mechanical ventilation | 217 (13%) | 159 (20.4%) | <0.001 | 13.5 | 149 (19.1%) | 159 (20.4%) | 0.525 | 2.2 |
| Site of Infection |  |  |  |  |  |  |  |  |
| Urinary Tract Infection | 491 (29.4%) | 191 (24.5%) | <0.001 | 15.2 | 88 (11.3%) | 67 (8.6%) | 0.299 | 10.7 |
| Pneumonia | 437 (26.1%) | 234 (30.0%) |  |  | 211 (27%) | 191 (24.5%) |  |  |
| Intra-abdominal Infection | 162 (9.7%) | 106 (13.6%) |  |  | 215 (27.5%) | 234 (30%) |  |  |
| Skin and soft tissues | 168 (10.0%) | 61 (7.8%) |  |  | 104 (13.3%) | 106 (13.6%) |  |  |
| Blood stream | 123 (7.4%) | 63 (8.1%) |  |  | 47 (6%) | 61 (7.8%) |  |  |
| Unknown source | 98 (5.9%) | 59 (7.6%) |  |  | 54 (6.9%) | 63 (8.1% |  |  |
| Others | 194 (11.6%) | 67 (8.6%) |  |  | 62 (7.9%) | 59 (7.6%) |  |  |
| Confirmed diagnosis of infection | 1266 (75.7%) | 643 (82.3%) | <0.001 | 12.4 | 617 (79%) | 643 (82.3%) | 0.096 | 5.9 |
| Inadequate antimicrobials | 527 (31.5%) | 125 (16%) | <0.001 | 29.1 | 149 (19.1%) | 125 (16%) | 0.110 | 5.5 |
| Mortality * | 179 (10.7%) | 104 (13.3%) | 0.059 | 5 | 105 (13.4%) | 104 (13.3%) | 0.941 | 0.2 |
| Hospital stay | 10 (6 – 16) | 11 (6 – 18) | <0.001 | 21.2 | 10 (6 – 17) | 11 (6 – 18) | 0.1530 | 7.5 |

The measurements for continuous variables are the median (IQR) and for categorical: n (%). SMD: standardized mean difference

***** The Mortality variable was not included to generate the propensity score.
