# Supplementary material for: Antimicrobials administration time in patients with suspected sepsis: is faster better? An analysis by propensity score
Source: J Intensive Care. 2020 Apr 22;8:28. doi: 10.1186/s40560-020-00448-1 (PMC7178597; doi:10.1186/s40560-020-00448-1)
Supplement: Supplementary file 3 — Additional file 3. Effect of the antimicrobial administration in the first hours of admission to the ED on in-hospital mortality. Models with total population. [file 40560_2020_448_MOESM3_ESM.docx]

**Additional file 3. Effect of the antimicrobial administration in the first hours of admission to the ED on in-hospital mortality. Models with total population**

|  | Antimicrobials ≤ one hour vs. > one hour | Antimicrobials ≤ three hours vs. > three hours |
| --- | --- | --- |
| All patients included | OR (95% CI)  n=2454 | OR (95% CI)  n=2454 |
| No adjustment | 1.13 (0.80; 1.6) | 1.28 (0.99; 1.66) |
| Adjusted for propensity score | 0.94 (0.63; 1.40) | 0.89 (0.67; 1.2) |
| Adjusted for covariates ^*^ | 0.98 (0,66; 1.45) | 1.04 (0.77; 1.40) |
| Adjusted for propensity score + covariates ^*^ | 0.89 (0,58; 1.37) | 0.87 (0.63; 1.22) |
| Patients with confirmed infection | n=1909 | n=1909 |
| No adjustment | 1.08 (0.75; 1.57) | 1.24 (0.93; 1.64) |
| Adjusted for propensity score | 0.99 (0.65; 1.53) | 0.92 (0.66; 1.28) |
| Adjusted for covariates ^**^ | 0.90 (0.59; 1.37) | 0.99 (0.72; 1.37) |
| Adjusted for propensity score + covariates ^**^ | 0.89 (0.55; 1.41) | 0.90 (0.63; 1.28) |
| Patients with adequate antibiotics | n=1802 | n=1802 |
| No adjustment | 1.06 (0.73; 1.56) | 1.24 (0.92; 1.66) |
| Adjusted for propensity score | 0.88 (0.57; 1.35) | 0.84 (0.59; 1.18) |
| Adjusted for covariates ^***^ | 0.86 (0.56; 1.32) | 0.97 (0.70; 1.36) |
| Adjusted for propensity score + covariates ^***^ | 0.79 (0.49; 1.27) | 0.83 (0.57; 1.21) |
| Patients with shock –Rivers | n=869 | n=869 |
| No adjustment | 0.87 (0.55; 1.36) | 1.09 (0.76; 1.56) |
| Adjusted for propensity score | 0.82 (0.48; 1.38) | 0.90 (0.59; 1.34) |
| Adjusted for covariates * | 0.82 (0.49; 1.37) | 0.96 (0.63; 1.44) |
| Adjusted for propensity score + covariates * | 0.75 (0.42; 1.34) | 0.86 (0.55; 1.35) |
| Patients with shock –sepsis 3 | n=179 | n=179 |
| No adjustment | 0.66 (0.33; 1.32) | 0.68 (0.36; 1.26) |
| Adjusted for propensity score | 0.53 (0.23; 1.20) | 0.54 (0.27; 1.09) |
| Adjusted for covariates * | 0.71 (0.32; 1.59) | 0.81 (0.38; 1.68) |
| Adjusted for propensity score + covariates * | 0.55 (0.22; 1.37) | 0.65 (0.29; 1.47) |

**^*^** Covariates: age, Charlson Index, intravenous fluids ≥ 1500 first hour, blood cultures in the first three hours, lactate, SOFA score, APACHE II score, confirmed diagnosis of infection, inadequate antimicrobials.

^**^ Covariates: age, Charlson Index, intravenous fluids ≥ 1500 first hour, blood cultures in the first three hours, lactate, SOFA score, APACHE II score, inadequate antimicrobials.

**^***^** Covariates: age, Charlson Index, intravenous fluids ≥ 1500 first hour, blood cultures in the first three hours, lactate, SOFA score, APACHE II score, confirmed diagnosis of infection.
